# The Hydrolytic Stability and Degradation Mechanism of a Hierarchically Porous Metal Alkylphosphonate Framework

**DOI:** 10.3390/nano8030166

**Published:** 2018-03-14

**Authors:** Kai Lv, Chu-Ting Yang, Yi Liu, Sheng Hu, Xiao-Lin Wang

**Affiliations:** Radiochemistry Lab, Institute of Nuclear Physics and Chemistry, China Academy of Engineering Physics, P.O. Box 919, Mianyang 621900, Sichuan, China; yangchuting@caep.cn (C.-T.Y.); arisewing@126.com (Y.L.); husheng205@caep.cn (S.H.); xlwang@caep.cn (X.-L.W.)

**Keywords:** metal-phosphonate frameworks, hierarchically porous materials, hydrolytic stability, degradation mechanism, radionuclide separation

## Abstract

To aid the design of a hierarchically porous unconventional metal-phosphonate framework (HP-UMPF) for practical radioanalytical separation, a systematic investigation of the hydrolytic stability of bulk phase against acidic corrosion has been carried out for an archetypical HP-UMPF. Bulk dissolution results suggest that aqueous acidity has a more paramount effect on incongruent leaching than the temperature, and the kinetic stability reaches equilibrium by way of an accumulation of a partial leached species on the corrosion conduits. A variation of particle morphology, hierarchical porosity and backbone composition upon corrosion reveals that they are hydrolytically resilient without suffering any great degradation of porous texture, although large aggregates crack into sporadic fractures while the nucleophilic attack of inorganic layers cause the leaching of tin and phosphorus. The remaining selectivity of these HP-UMPFs is dictated by a balance between the elimination of free phosphonate and the exposure of confined phosphonates, thus allowing a real-time tailor of radionuclide sequestration. Moreover, a plausible degradation mechanism has been proposed for the triple progressive dissolution of three-level hierarchical porous structures to elucidate resultant reactivity. These HP-UMPFs are compared with benchmark metal-organic frameworks (MOFs) to obtain a rough grading of hydrolytic stability and two feasible approaches are suggested for enhancing their hydrolytic stability that are intended for real-life separation protocols.

## 1. Introduction

The insufficient chemical, thermal or mechanical stability of metal-organic frameworks (MOFs) under humid vapor, acidic/basic liquor, toxic chemicals or other critical environments remains a bottleneck for their large-scale application in storage, delivery, separation, transformation and detection of target species, despite ever-increasing reports of “stable” MOFs since the last decade [1,2,3]. Many have investigated fabricating novel porous coordination polymers (PCP) with controlled stability by tuning metal-ligand bond strength, chemical functionality of linkers or by regulating framework architectures, surface hydrophobicity [4,5]. A compositional utilization of highly charged cations (e.g., Zr^4+^) and pK_a_ of ligands (e.g., carboxylate) has served as an exemplary construction of robust MIL (Materials of Institute Lavoisier), UiO (University of Oslo) MOFs that recently extended to radionuclide sequestration due to appreciable hydrolytic stability and accessible functionality [6,7]. In fact, it becomes arguable that these benchmark MOFs are hydrolytically stable in an acidic digestion solution since taking polycrystallinity or surface area as proof of stability lacks quantitative insight into degradation mechanisms. 

An equivalent to these carboxylate MOFs—metal-phosphonate frameworks (MPFs)—has emerged as promising radionuclide separation materials, based on their tunable porosity and miscellaneous linker functionality [8,9,10]. Most importantly, the presumable high level of hydrolytic stability of MPFs stems from their strong tetravalent metal-oxygen-phosphorus bonds and interconnected framework architecture. However, these MPFs remain less explored due to a lack of crystallinity and are designated as unconventional MOFs or unconventional metal-phosphonate frameworks (UMPFs) [11]. Microporous UMPFs are capable of separating trivalent lanthanides from actinyl at pH ~ 2 binary media [12]. Single-crystal MPFs have been laboriously prepared and sequester uranyl efficiently from pH ~ 1 solution [13]. Normally, UMPFs and MPFs exhibit superior imprisonment of radionuclides from an acidic digestion solution over carboxylate MOFs. However, exploring the driving forces behind the instability/stability of UMPFs and MPFs is in its infant stages, and is particularly underexplored in harsh acidic media.

Hierarchically porous UMPFs (HP-UMPFs) had introduced a set of mesopores/macropores that readily provide accessible selectivity and lend their uses to miscellaneous applications compared with microporous counterparts [14]. Tin and niobium-based HP-UMPFs have demonstrated excellent enrichment of Th^4+^, UO_2_^2+^ or lanthanides from radionuclide surrogates, which hold great potential for radioanalytical separation [15,16]. However, hierarchically porous materials have large pore voids that are intrinsically metastable, leading to pore size enlargement and structural stability rarely being achieved together [17]. While near total disorder of these HP-UMPFs is hardly amenable to precise microstructure elucidation, they induce active sites accessible to both target radionuclides and corrosive molecular liquids, creating a compromise between binding selectivity and structural stability [18]. To date, the hydrolytic stability of polyphosphonate-functionalized mixed oxides has been assessed for their usefulness in nuclear applications [19,20]. However, to the best of our knowledge, the quantitative assessment of the chemical and thermal stability of HP-UMPFs has never been reported. Hence, the investigation of the level of hydrolytic stability of HP-UMPFs becomes imperative under experimental close to radioanalytical separation that frequently involves strong inorganic acids and redox agents. 

In this contribution, we investigated the chemical and thermal stability of tin (IV) alkylphosphonates as an archetypical HP-UMPF both from the bulk phase and the surface structure. The effect of acidity and temperature on the degradation of hydrochloric acid will be explored and the kinetic stability of the bulk phase will also be evaluated to determine whether the degradation continues or whether it plateaus to a stable state. The variation of particle morphology, hierarchical porosity, backbone composition and thermal stability will complement the surface structure transformation. Furthermore, the remaining sequestration selectivity will contribute to uncovering a plausible degradation mechanism for these HP-UMPFs. They will also be compared with benchmark MOFs and UMPFs, shedding light on the judicious fabrication of HP-MPFs with elevated stability aimed at real-life radioanalytical separation. 

## 2. Material and Methods

### 2.1. Sample Preparation and Characterization

The pristine hierarchically porous tin (IV) alkylphosphonates (SnP) were synthesized according to a previously described method [15]. By adopting alkylphosphonate with a different number of –PO_3_H_2_, they are denoted as Sn-HEDP (bi-phosphonic), Sn-ATMP (tri-phosphonic), Sn-EDTMP (tetra-phosphonic) and Sn-DTPMP (penta-phosphonic). For each type, 40 mL 3 mol·L^−1^ HCl was poured into clean polypropylene vials containing 80 mg sample, then dispersed in air-bathed orbital shaker at 303 K for 1 day. The slurries were filtered and washed with deionized water until neutral pH, dried overnight at 383 K and then placed at desiccator prior to analysis. 

The N_2_ adsorption-desorption isotherms of each pristine and leached sample were measured using Micromeritics 3Flex sorption analyzer (Micromeritics, Norcross, GA, USA), in which the Brunauer-Emmett-Teller (BET) surface area was determined in a relative pressure range of 0.15~0.20 and the total pore volume was measured at P/P_0_ = 0.95 (degas at 120 °C for 4 h). The X-ray photoelectron spectroscopy (XPS) spectra of each element were recorded using Thermo ESCALAB 250Xi spectrometer (Thermo Fischer, Waltham, MA, USA). High-resolution field-emission scanning electron micrographs (FE-SEM) were obtained using Carl Zeiss Ultra55 (Carl Zeiss GmbH, Oberkochen, Germany) with gold coating. Transmission electron microscopy (TEM) was performed on FEI Tecnai G2F30 (FEI, Hillsboro, OR, USA). Thermogravimetric-differential scanning calorimetric analysis was performed using SDT Q600 (TA instruments, New Castle, PA, USA) at a heating rate of 10 °C /min to 1000 °C under air. The Fourier Transform Infrared Spectroscopy (FT-IR), Raman spectra were recorded at Nicolet Magna FTIR spectrometer (Nicolet, Waltham, MA, USA) and LabRAM HR800 micro-Raman spectroscopy (HORIBA, Kyoto, Japan), respectively. The ^31^P MAS-NMR spectra were measured at Bruker Avance III 400 (Bruker, Billerica, MA, USA).

### 2.2. Leaching and Uptake Test

The leaching of SnP was undertaken in various hydrochloric acid media in order to evaluate the effects of two physicochemical parameters including the acidity (0.01, 0.1, 1.0, 3.0 mol·L^−1^) and temperature (303, 318, 333 K). Meanwhile, the dissolution kinetic was obtained in terms of leaching duration (0.5–168 h). For each leaching test without leachate renewal, 10 mg of sample was put in contact with 40 mL of a certain hydrochloric acid at a certain temperature (see Figure 7 caption) in the 50 mL centrifuge tube. At given intervals, each tube containing slurry was centrifuged at 8000 rpm for 5 min in order to eliminate any colloids and 5 mL supernatant was collected. 

The selectivity experiments are carried out using surrogate solutions containing Cs^+^, Sr^2+^, Al^3+^, Fe^3+^, Th^4+^, UO_2_^2+^, Ln^3+^ (without Pm), prepared from standard solutions (Plasma, Cal, SCP Science, Quebec, QC, Canada). 20 mL of simulant solution (0.1 mol·L^−1^ HNO_3_) with an initial concentration of 3.5 mg L^−1^ for each element tested is pipetted into a 45 mL centrifugal tube loaded with 10 mg tin phosphonate, and then the tubes are placed in the WIGGENS WS-300R orbital shaker (T = 30 °C, rpm = 260) (WIGGENS, Berlin, Germany) for 24 h. After being centrifuged at 7000 rpm for 4 min, 5 mL supernatants are obtained. The element concentrations of both categories of samples were determined by inductively coupled plasma-optical emission spectroscopy (Agilent Technologies 700 Seires, Agilent, Santa Clara, CA, USA). The distribution coefficient (Kd, mL∙g^−1^) and separation factor (SF, a.u.) are defined as previously described [16].

### 2.3. Determination of the Normalized Leaching and Dissolution Rates

The leachability of element *i* (*i* = Sn, P) is described by the normalized leaching *N_i_* (gm^−2^) and normalized dissolution rate *R_i_* (gm^−2^·d^−1^) according to the following equations [21]:

(1) $$N_{i}=\frac{m_{i}}{\omega_{t}\times S}$$

*R_i_* = *N_i_*/*t* (2)

where *m_i_* the total amount of *i* measured in solution (g), *S* the effective surface area (assuming the BET surface area fully accessible, m^2^) of the sample in contact with the solution, *ω_t_* is the mass ratio of the element *i* in the solid and *t* corresponds to the leaching duration. The dissolution was qualified as congruent if all elements were released with the same ratio as pristine stoichiometry [22]. A direct comparison of *R*_Sn_ and *R*_P_ values was made through the determination of the congruence ratio—*r* = *R*_Sn_/*R*_P_.

## 3. Results and Discussion

**Figure 1:**
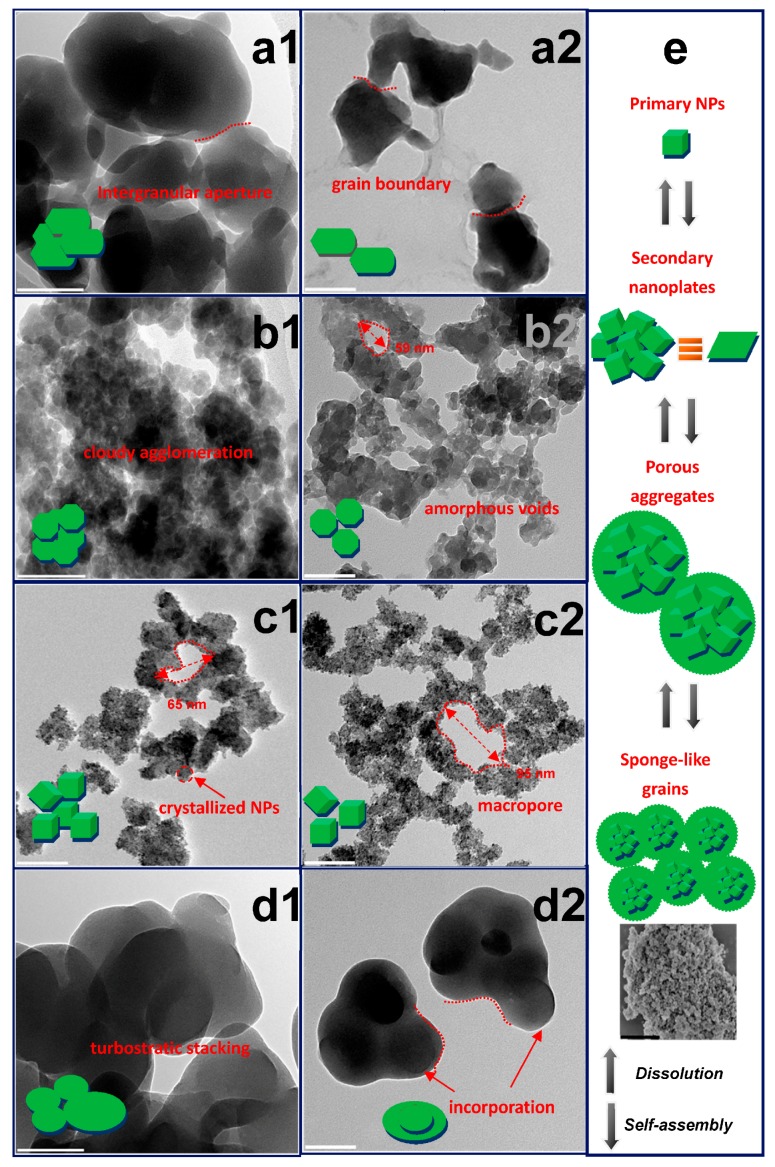
The particle morphology transformation illustrated by Transmission electron microscopy (TEM) images: (**a**) Sn-HEDP; (**b**) Sn-ATMP; (**c**) Sn-EDTMP; (**d**) Sn-DTPMP. The **a1**–**d1**, **a2**–**d2** corresponds to the pristine and leached tin alkylphosphonates, respectively. The scale bar is 100 nm; (**e**) Schematic drawing of triple-level SnP nanoparticle arrangement into hierarchical porous materials and the disassembly process. The SEM image of pristine Sn-EDTMP has the scale bar of 2 μm.

Prior to the revelation of structure transformation, self-assembly of archetypical HP-UMPFs can be proposed based on previous structural models [23,24]: (i) two anionic P–O groups or one neutral P=O group of each type of alkylphosphonate acids would strongly coordinate with tin(IV) in various bridging modes. These SnO_6_ octahedral and PO_4_ tetrahedral form the 1D polymeric units; (ii) nonrigid alkyl pillars connect these units, making each of the uneven layers and generating intralayer channels or microporous voids; (iii) these layers stack in turbostratic disorder and induce a certain amount of unbounded phosphonic acid groups between layer gaps statistically; (iv) the house-of-cards arrangement of nanolayers aggregates along the template micelles, forming wide-distributed mesopores after soft-template removal. The meso-particles progressively aggregate into even larger sponge-like macroporous grains (Figure 1e). Conversely, each hierarchical porous structure of tin alkylphosphonate is supposed to degrade in rough sequences.

### 3.1. The Variation of Hierarchical Porous Morphology Induced by Corrosions

**Figure 2:**
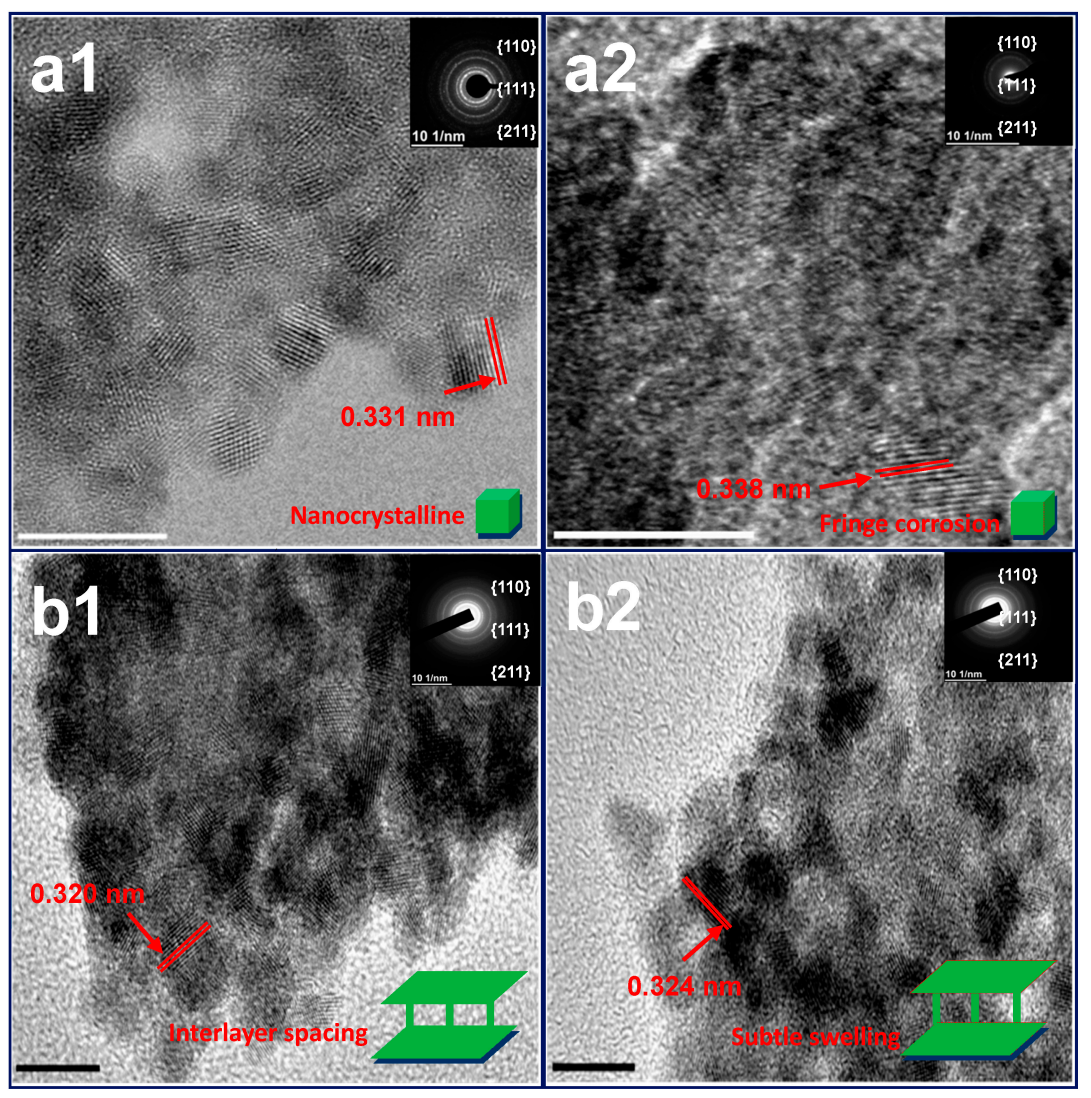
The subtle variation of Sn-EDTMP nanoparticles is revealed by high-resolution TEM images and electron diffraction patterns. Samples a,b are Sn-EDTMP5 and Sn-EDTMP3. The (**a1**,**b1**), (**a2**,**b2**) corresponds to the pristine and leached Sn-EDTMP, respectively. The electron diffraction patterns have been calibrated as inset and the scale bar is 5 nm.

Both pristine Sn-HEDP and Sn-DTPMP display dense multilayer nanostructures with diameters of a few hundred nanometers. These compact aggregates are prone to disassembly into irregular fractures along interlaced grain boundaries while the dissociative products retain turbostratic layer stacking upon leaching (Figure 1a,d). Rarely, the large spheroidal Sn-DTPMP nanoparticles (NPs) with micrometric pores appear to have incorporated tiny moieties during dissolution. This phenomenon was presumably driven by the minimization principle of surface free energy of inorganic layers, which hold abundant metastable defects and create active external surface areas as previously reported niobium(V) alkylphosphonates [16]. The Sn-ATMP demonstrates dense agglomeration with amorphous voids in the meso regime and their intraparticle apertures evolve into atypical cavities concurrent with the detachment of secondary nanoplates upon corrosions (Figure 1b). The uniform-sized Sn-EDTMP NPs, with a few nanometers in diameter, have been wrapped by an amorphous region (Figure 1c). During leaching, these hybrid aggregates become decentralized to generate larger macropores (Figure 1c2, Figure S1). These clear lattice fringes of nanobeam diffraction patterns, indexed to a primitive-cubic topology (Figure 2), have an interlayer spacing (3.2~3.3 Å) of the nanocrystalline segments and accord with the X-ray Diffraction (XRD) results (Figure S7). They become blurred and expand to a subtle extent upon acidic attack, indicating a minor layer swelling. The variation of preceding morphology attests that dissolution of these HP-UMPFs can be roughly regarded as a continuous degradation of porous phases [25].

**Figure 3:**
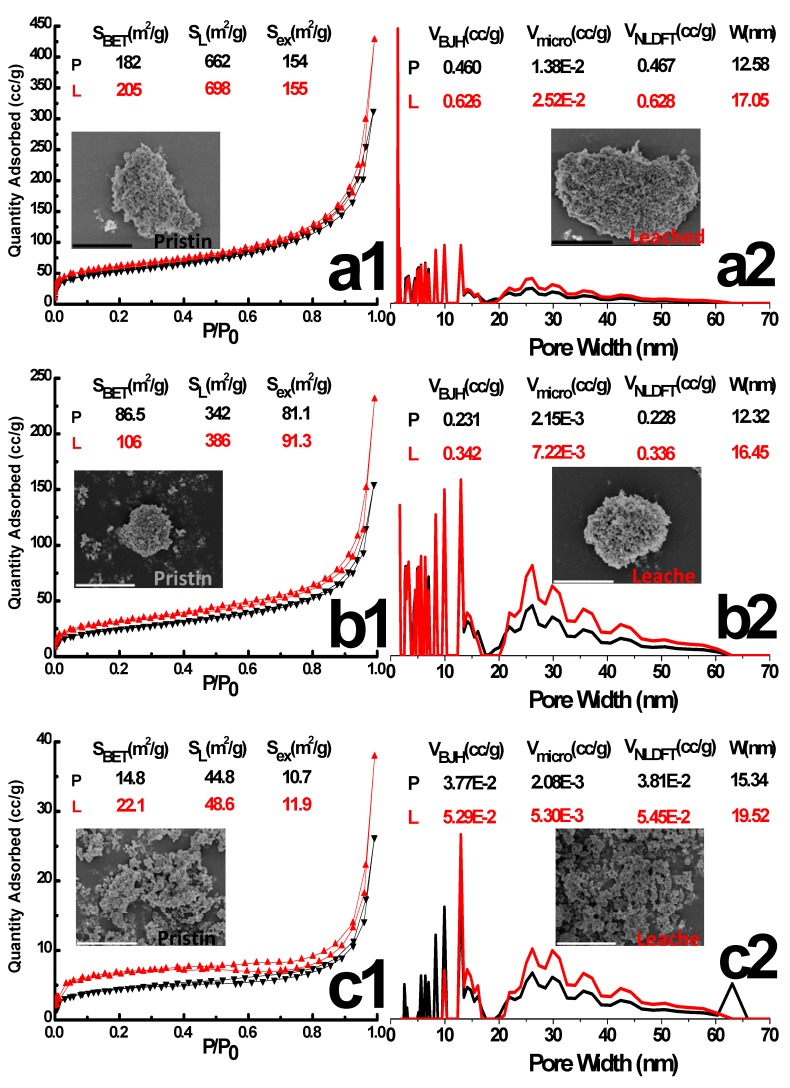
The N_2_ adsorption-desorption isotherm and Pore Size Distribution (PSD) of pristine (P) and leached (L) tin alkylphosphonates by NLDFT: (**a**) Sn-ATMP; (**b**) Sn-EDTMP; (**c**) Sn-DTPMP. The surface area, pore width, pore volume and particle morphology are depicted as inset. The scale bar of SEM image is 5 μm. The S_BET_, S_L_, S_ex_, V_BJH_, V_micro_, V_NLDFT_ and W in the figure represents BET surface area, Langmuir surface area, external surface area, BJH mesopore volume, BJH micropore volume, NLDFT micropore volume and BJH pore width

The N_2_ adsorption-desorption isotherms of pristine, leached samples are the IV/I hybrid type with H3 hysteresis (Figure 3), which indicates a disordered wormlike mesoporous structure. Leaching in 3 mol·L^−1^ HCl for 24 h neither altered the overall isotherm shape nor the pore size distribution (PSD) dramatically, although it resulted in a 13%, 22%, 49% increase in the total surface area for Sn-ATMP, Sn-EDTMP and Sn-DTPMP, respectively. Since internal surface area of three-level hierarchical porosity solely contributes to the microporous volume, an approximate 2-fold increase of internal surface area implies that the acidic leaching will readily reopen microporous spaces by eliminating free phosphonates linkers that previously hinder inside channel regions. Additionally, an increase of mesoporous volume and a shift of PSD to larger diameter region collectively hint bulk corrosions occur along the grain boundary and crack the intraparticle apertures. The meso-, macropore hierarchy will offer accessible environment for radioactive species and corrosive acids, rendering the external surface area equivalent to energetically reactive sites due to an ultrasmall portion of microporosity. Overall, the integrity of trimodal porosity reveals that these HP-UMPFs are hydrolytical resilient and will tolerate extreme conditions without suffering great degradation of porous texture, albeit each level of porosity underwent a degree of modification by aqueous corrosion.

### 3.2. The Alteration of Skeletal Composition Caused by Leaching

**Figure 4:**
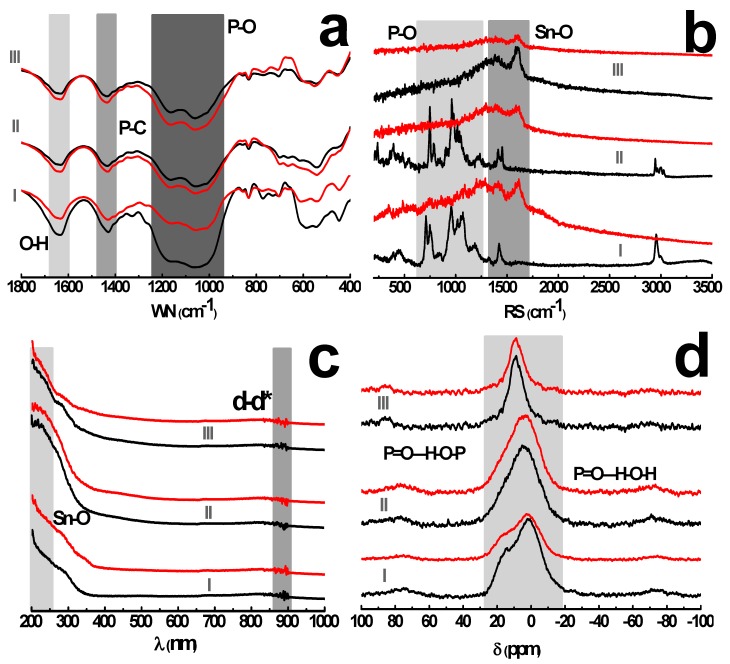
The backbone composition of pristine and leached tin alkylphosphonates: (**a**) Fourier Transform-Infrared (FT-IR) spectra; (**b**) μ-Raman spectra; (**c**) solid UV-Vis spectrum; (**d**) ^31^P spectrum. The Sn-ATMP, Sn-EDTMP and Sn-DTPMP are represented by I, II and III, respectively. The WN, RS represents wave number, Raman shift, respectively.

The spectra of tin alkylphosphonates demonstrate a characteristic stretching vibration of P–C at 1440 cm^−1^ and a bending vibration of O–H from phosphonate moiety or structural water at 1640 cm^−1^ (Figure 4a). The strong adsorption band, between 950 and 1250 cm^−1^, pertains to the stretching vibration of P–O bonds. Besides, the bands in the region between 700 and 850 cm^−1^ are attributed to C–H bending vibrations from the methylene in the alkyl chain. The Sn–O vibration occurs in the region below 600 cm^−1^. The typical bands of P–O, P–C and –CH_2_– are visible on both spectra of pristine, leached samples while a significant attenuation of P=O vibration around 1100–1200 cm^−1^ is observed for leached Sn-ATMP, which indicates that leached sample liberates certain amount of phosphonate ligands into bulk solution while retains free phosphonic acid groups. It appears that Sn-ATMP decomposes into lower hierarchical aggregates as described in Figure 1e. The Raman spectra as complementary to FT-IR spectra (Figure 4b) show that the bands around 709–1069 cm^−1^ of pristine Sn-ATMP, Sn-EDTMP are attributed to the stretching vibration of P–O bonds while their bending modes are located in the region below 500 cm^−1^. The medium peaks at 1422 cm^−1^ for Sn-ATMP, 1454 cm^−1^ for Sn-EDTMP and 1590 cm^−1^ for Sn-DTPMP are assigned as in-plane deformation vibration of Sn–O cluster. The surface metal-ligand bonding deteriorates, evidenced by the disappearance of P–O bands and the broadening of Sn–O regions. The spectra variations collectively imply that the nucleophilic attack occurs heavily on the Sn–O–P bonds.

The solid UV-Vis spectra (Figure 4c) demonstrates an intense adsorption region around 200~250 nm, assigned as a strong charge transfer from coordinating oxygen to tin centers. The minor adsorption band between 870 and 897 nm corresponds to d-d* electronic energy level transitions in distorted coordination field. Both adsorption regions exhibit indiscernible change upon leaching, which implies the coordination mode of these building units remains largely intact. The ^31^P MAS-NMR spectra of acid-washed sample have more resonances towards higher chemical shift, which indicates multiple de-shielding environments arise from the breakdown of inorganic layers, especially for Sn-ATMP (Figure 4d). The low chemical shift between −1 and 15 ppm is attributed to multiple phosphorus species in turbostratic layers [9], where the hydrogen bonds are omnipresent derived from the neighboring phosphonic acid groups or layer-resided water molecules in the genre of P=O—H–O–P or P=O—H–O–H (P=O as acceptor and P–OH or H_2_O as donor). Moreover, the zwitterionic amines are probably involved in alternative intramolecular O–H—N–C hydrogen bond, which resembles strategic O–H—N=C enhancing the chemical stability of 2D porphyrin COFs [26]. The ^31^P decrease in relative intensity of Sn-ATMP shows an elimination of its P species. These hydrogen-bonding interactions will facilitate the protection of alkyl pillars and phosphonate moieties from hydrolysis in the presence of hydrochloric acid.

The methylene C 1s peak of the XPS spectrum was corrected at 284.8 eV to eliminate the differential charging upon photoionization. The corresponding energetically corrected spectra of Sn 3d, P 2p, O 1s and N 1s all shift to lower binding energy (BE) due to the compositional leaching of each element that impairs the shielding environment and facilitates the escape of valence electron (Figure S3). Previous degradation of mesoporous silica occurred via two-step nucleophilic attack [27]. Similarly, we propose the reactions as: the first is the hydrated protons locates on the defective metal center and stretch the Sn–O–P bonding via electron-withdrawing effect; the second is the chloride ions subsequently impairs the bondage until it breaks. Thus, the tin chlorides are readily formed and the liberated hydrated protons will coexist with negative alkylphosphonate ligands. Besides, an increase in the relative weight percentage of surface tin, phosphorus of leached Sn-ATMP, Sn-EDTMP manifests a considerable degradation of N-bearing alkyl moieties. These degradation products can be identified by comparing each core level of leached elements with standard spectra (Figure S2). 

### 3.3. The Thermal Stability of Pristine and Leached HP-UMPFs

**Figure 5:**
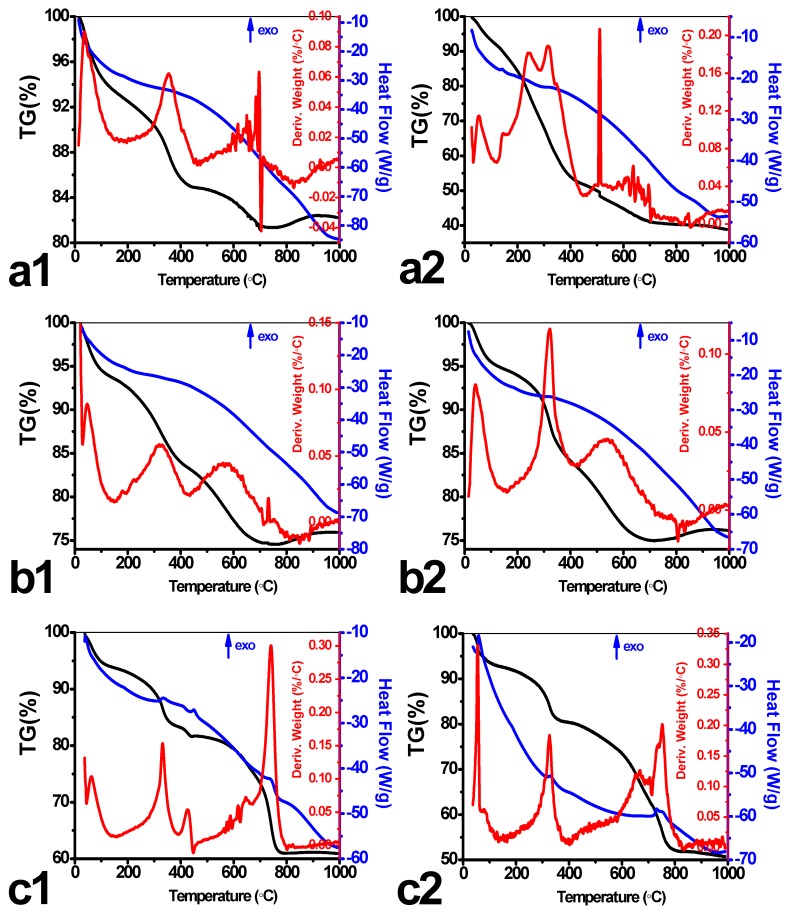
The TGA-DSC profiles of pristine (**a1**–**c1**) and leached (**a2**–**c2**) tin alkylphosphonates: (**a**) Sn-ATMP; (**b**) Sn-EDTMP; (**c**) Sn-DTPMP.

The representative thermogravimetric patterns of pristine, leached HP-UMPFs in Figure 5 manifest a gradual weight loss of adsorbed solvent through the temperature interval of 25–200 °C. Following the water evaporation, a broad exothermic peak centered at ~320 °C is ascribed to the progressive decomposition of alkyl pillar moieties with the emission of CO_2_, NO_2_. The approximate thermal stability temperature (T_d_) of leached Sn-ATMP is determined as 510 °C while the pristine is ~640 °C. Given that a significant loss of free phosphonate ligands occurs upon leaching, the variation of T_d_ indicates that HP-UMPFs endowed with unbounded phosphonate linkers are more thermally stable than their pure counterparts. The nanocrystalline Sn-EDTMP and its leached sample degrades until ~590 °C and displays almost identical weight loss, suggesting that a local atomic order affords the enhancement of thermal or hydrolytic stability. Both pristine and leached Sn-DTPMP has the T_d_ ~ 710 °C and the total weight loss is 38% and 47%, respectively. The unexpected high T_d_ depends largely on the intense layer crosslinking, although these N-bearing alkyl pillars are more susceptible to thermolysis since nitrogen atoms attenuate neighboring C–C bonds by electron withdrawing [28]. It is reported that the final decomposition product of tin arylphosphonates is SnP_2_O_7_ until 1000 °C [9], shedding light on the immobilization of adsorbed long-lived radionuclides in ceramic wasteforms. In this sense, a relatively low thermal stability appears favorable as it becomes more facile to transform these UMPFs to pyrophosphates via pyrolysis. 

### 3.4. The Remaining Functionality in Relevant to Pristine Sequestration Selectivity

**Figure 6:**
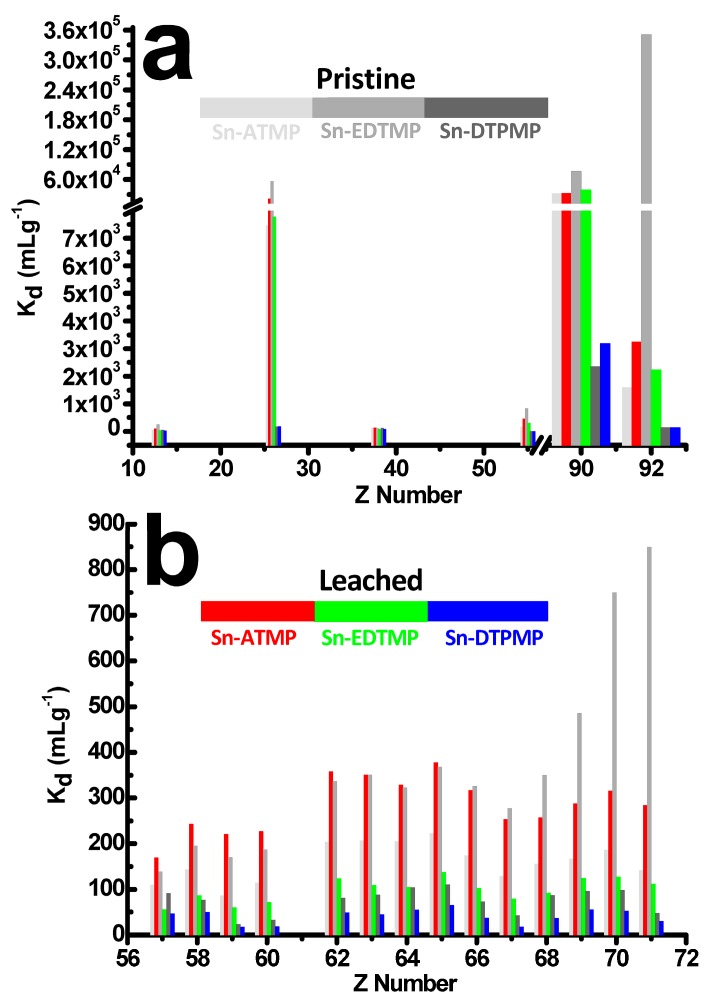
The selective uptake profiles of pristine and leached SnP in radionuclide surrogate bearing 20 elements: (**a**) K_d_ values for alkali, alkaline earth, transitional, light actinides (**b**) detailed K_d_ values for lanthanides.

The ultimate defective Sn-ATMP and Sn-DTPMP provide near total short-range disorder yet its function is a somewhat ordered, which follows the charge discrimination effect [11]. Specifically, they exhibit high affinity for Th^4+^, Fe^3+^ while repulsing trivalent lanthanides, Al^3+^, Sr^2+^, Cs^+^ (Figure 6a). Upon corrosions, the leached Sn-ATMP demonstrates an unexpected larger uptake of each cation, leading to an approximately 2-fold increase in lanthanide uptake (Figure 6b) yet an extinct selectivity for Th^4+^, in which the SF_Th/Fe_ decrease from 4.2 to 1.5. The exceptional uptake of uranyl dramatically diminishes to the K_d_ value of 2228 mg L^−1^ in relative to 350,808 mg L^−1^ for pristine Sn-EDTMP, in which the SF_Th/Fe_ increase from 1.4 to 5.0 and retains lanthanide uptake patterns in conformity with the ionic radius contraction. Likewise, the leached Sn-DTPMP has lower K_d_ for each cation but a larger SF_Th/Fe_ = 19 in relevant to the pristine.

Similar to the layered-like crystalline zirconium alkylphosphonate, the tin alkylphosphonates are presumably equipped with dual cationic binding sites: (i) free phosphonates bearing one anionic P–OH and one neutral P=O group that located on the external surface areas; (ii) confined phosphonates containing one anionic P–OH in the channel region that used to form hydrogen bond with structural water. It is postulated that the free phosphonates will favorably bind with Th^4+^ due to the relatively high positive charge density while steric effect endows the confined phosphonates with preferential uptake of Fe^3+^. If the exposure of confined phosphonates by uncovering the layer spacing outnumbers an elimination of free phosphonates upon corrosion, the sequestration affinity will increase yet the SF_Th/Fe_ decreases. In other words, a dynamic balance between the elimination of free phosphonates and the exposure of confined phosphonates will dictate the sequestration affinity towards radionuclides of a different charge density. If the amount of these two types is feasibly controlled, one can efficiently tune cation selectivity towards radionuclides of high charge. It is reasonable that acid corrosion will first eliminate surface-bounded free phosphonate moieties, followed by deteriorating inorganic layers that enables radionuclides to enter these confined binding sites. Meanwhile, the subtle swelling of interlayer spacing commences to facilitate the transportation of exotic species and those organic pillars become more susceptible to degradation. 

### 3.5. Effect of Acidity, Temperature on Dissolution Rates and Kinetic Stability

**Figure 7:**
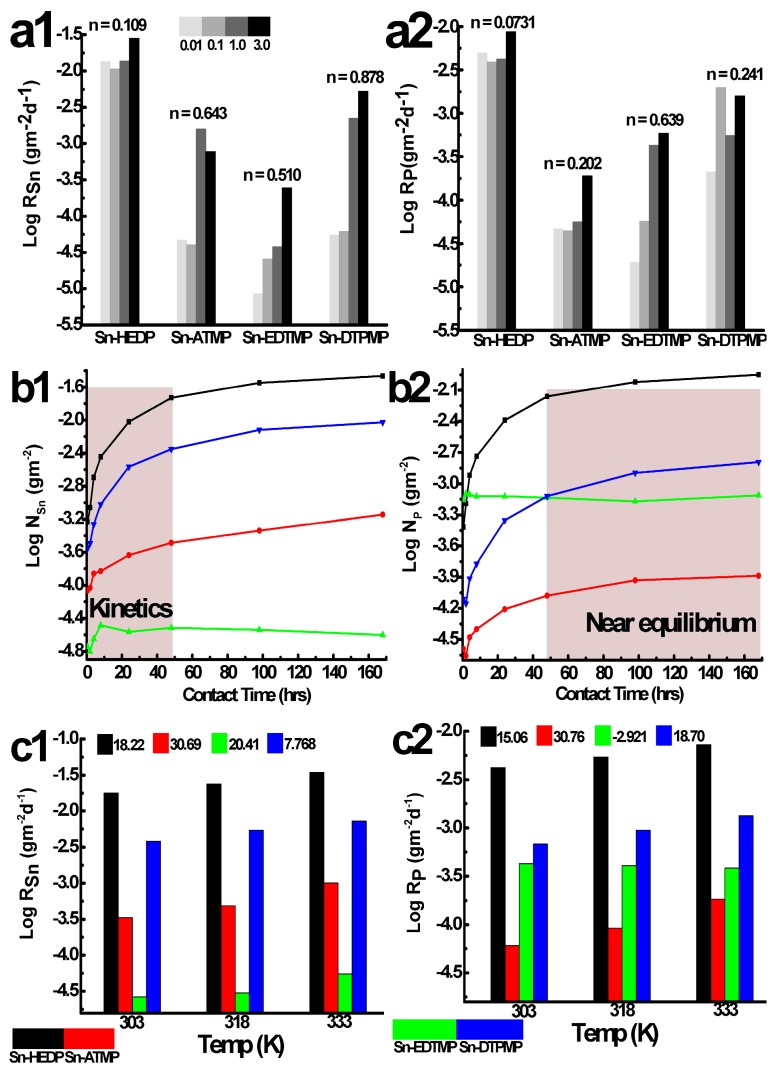
The batch chemical stability of tin alkylphosphonates in hydrochloric acid: (**a**) the effect of acid concentration (303 K, 2 days); (**b**) the hydrolytical kinetics ranging from 0.5 to 168 h (1 mol·L^−1^, 303 K); (**c**) the effect of solution temperature (1 mol·L^−1^, 2 days). The **a1**–**c1**, **a2**–**c2** corresponds to the element tin, phosphorus, respectively.

Due to the unequivocal significance of acidity on the macroscopic dissolution behavior, the effect of acidity on the normalized dissolution rates had been conducted between 10^−2^ and 3 mol·L^−1^, falling into the concentration range (4~10 mol·L^−1^) that the radioanalytical separation protocols frequently adopt. The leaching time is 24 h and the rendering of the degradation occurs far from thermodynamic equilibrium and allows accurate determination of *R_i_*. The dissolution pattern of tin is consistent with phosphorus for Sn-HEDP, displaying an initial drop followed by a gradual increase in the whole acidity range (Figure 7a). It attributes to the actual decrease in proton concentration since it prefers to acidify the defective metallic sites and deteriorate Sn–O–P layers with the aid of Cl^−^ and OH^−^. The elemental dissolution of Sn-EDTMP positively correlates with the increase of acidity, reaching its maximum R in the magnitude of 10^−3^ gm^−2^·d^−1^ in 3 mol·L^−1^ HCl. The dissolution pattern of tin in Sn-DTPMP and phosphorus in Sn-ATMP resemble the general trend of Sn-HEDP and Sn-EDTMP, respectively. However, tin in Sn-ATMP and phosphorus in Sn-DTPMP have largest dissolution rate of 0.00158, 0.00198 gm^−2^·d^−1^ at 1.0, 0.1 mol·L^−1^ HCl, respectively. The competitive effect of ionic strength and acid concentration illustrates for the ever-reducing stability. The large variation of partial order coefficient n (Figure S4, 0.0731~0.878) suggests a distinct proton attack degree among these tin alkylphosphonates [29]. The total *R*_p_ including P species either from hinder phosphonates or free phosphonates has been determined as low as 10^−2^~10^−4^ gm^−2^·d^−1^, partially evokes its incongruence with *R*_Sn_ (Table S1), which ranges from 10^−2^ to 10^−5^ gm^−2^·d^−1^. 

The leaching of these HP-UMPFs does not appear to be heavily affected by the temperature since the *R* increases by less than a factor of 3, ranging from 303 to 333 K (Figure 7c). The fitting results from the Arrhenius equation demonstrate that the dissolution constants of tin, phosphorus follow the order: Sn-ATMP > Sn-HEDP > Sn-DTPMP > Sn-EDTMP (Figure S6). It is worth noting that the hydrolytically stable Sn-EDTMP fails to fit into this equation (*R*^2^ = 0.72) and the dual regional P species reached a lower saturation at higher temperatures. The calculated activation energy is relatively low and suggests the macroscopic dissolution occurring at the solid-liquid interface is likely controlled by species diffusion [30]. 

The similar tendency in the variations of tin, phosphorus dissolution during kinetic stability can be divided into two regimes for Sn-HEDP, Sn-ATMP and Sn-DTPMP (Figure 7b): the first is rapid leaching in the first two days, during which both elements are quickly released from the bulk phase. The second is near the equilibrium procedure that partially leached species reversibly accumulate on the corrosion pits, alleviating overall dissolution rates and inducing incongruence simultaneously. The dissolution kinetics is determined by neoformed phase solubility and the concentration of leached species [31]. The fitting results in Figure S5 reveal that the dissolution kinetics of Sn-HEDP and Sn-ATMP are in agreement with the first-order model and yield a rate constant value in the order of 10^−2^ d^−1^. The dissolution data of Sn-DTPMP can be well interpreted by a parabolic model (*R*^2^ > 0.99). The amorphous shells of Sn-EDTMP degrades fairly rapidly in the first regime, afterwards it slowly approaches near saturation, yielding a mean rate of 3.58 × 10^−6^ gm^−2^·d^−1^ for tin, 1.10 × 10^−4^ gm^−2^·d^−1^ for phosphorus over 7 days. Since local atomic ordering normally confers resistance to an acidic attack, it takes a long time for the nanocrystalline region of Sn-EDTMP to degrade in contrast with amorphous Sn-ATMP and Sn-DTPMP, demonstrated by the apparent kinetic discrepancy. 

### 3.6. Degradation Mechanism and Implication for Radioanalytical Separation

**Scheme 1:**
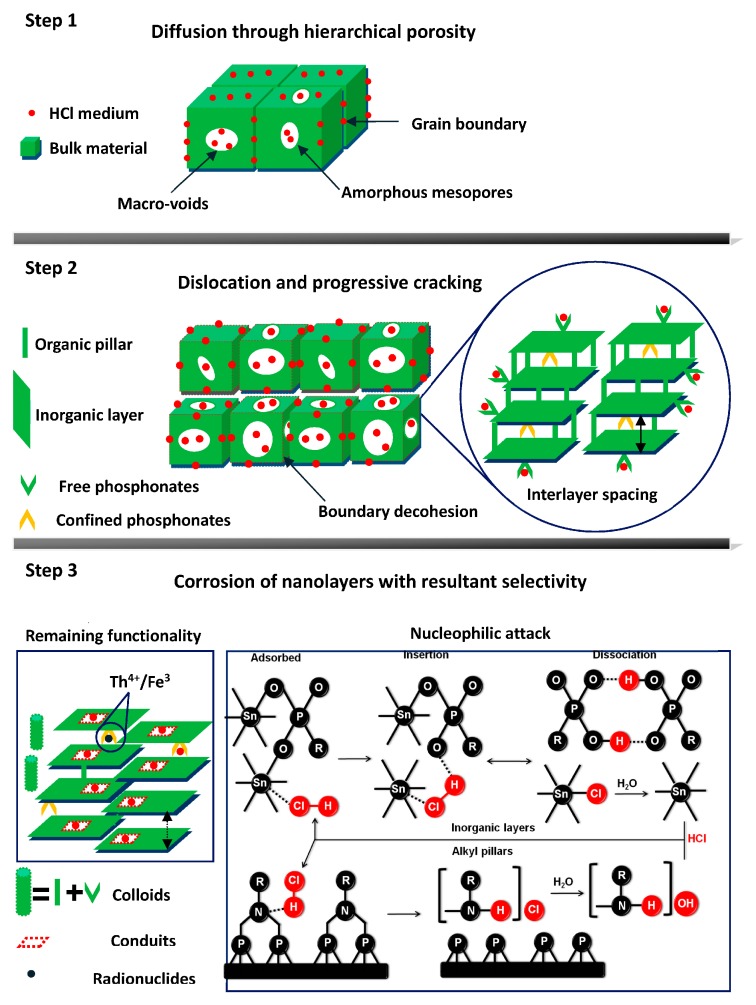
A plausible degradation mechanism of hierarchically porous tin alkylphosphonates. The intramolecular hydrogen bonds are omitted for the sake of clarity.

It will be reasonable to propose the degradation as a three-level disassembly of monolith materials (Scheme 1). The first step of the dissolution process is a rapid diffusion of hydrated protons, chloride or hydroxyl ions into macro-voids or amorphous mesopores along the grain boundary, while the surface active sites will adsorb these cations and anions via electrostatic interaction. Secondly, the surface-adsorbed nucleophiles gradually dissolve intergranular species and induce large aggregates to crack into sporadic fractures. Meanwhile, free phosphonate ligands are liberated together with a certain amount of alkyl pillars at the edge of inorganic layers, in which period the penetrated corrosive ion pair commences generation of the corrosion conduits on the nanolayers. Once the fractional nanolayers break up, the intrusion of the acidic medium containing radionuclides will continually deteriorate the alkyl pillars and expose the confined phosphonate ligands. These degraded alkyl fragments are mixed with liberated tin species or phosphate moieties to generate hybrid colloids as the interlayer spacing swells until totally eliminated. The nucleophilic reaction comprises: (i) irreversible insertion of HCl, in which the chloride anion will bond with tin while the displaced oxygen from the linker still participates in a H-bond; (ii) the tin chloride complexes will irreversibly hydrolyze, condensate into hydroxide species and the phosphonate acids are able to form a dimer structure. The N-bearing alkyl pillars will somehow protect the phosphonate groups from HCl through the formation of the –RN^+^(CH_2_)_2_Cl^−^ adduct.

The previous degradation mechanism of some MOFs underlined the significant effect of metal-ligand bond strength, neighboring atoms and metal inertness on their thermodynamic stability [28,32,33] while the resistance of mesoporous silica to mineral acids was highly dependent on a wall thickness-pore diameter threshold [27]. The strong Zr–O–P bond strength endows Zr-based MPFs with outstanding stability [34]. In the case of these HP-UMPFs, the Sn–O–P bond strength is moderate, while electron withdrawing nitrogen atoms compensate for the instability of tin centers that readily form complexes with chloride. The large proportion of meso/macro volume is relative to microporous regimes indicative of low wall thickness-pore diameter threshold appears vulnerable to acid stress while giant meso/macro porosity would first collapse followed by deteriorating micropores.

So far, the use of bi-, tri-alkylphosphonate ligands to construct tin alkylphosphonate frameworks cannot offer satisfactory hydrolytic stability while the utilization of penta-alkylphosphonate ligand has been discouraged by their tendency in intense layer crosslinkages leading to poor accessibility. Thus, the linking of tetra-alkylphosphonate acid or monoesters with metal(IV) clusters will be a good choice in fabricating hydrolytically stable HP-UMPFs without compromising ion selectivity. Indeed, the intrinsic stabilities of tin-based HP-UMPFs are comparable to benchmark carboxylate MOFs, MPFs, microporous UMPFs (Table S2) and are at least ranked as “4A” given the harshness of exposure and retention of hierarchical porosity [35]. Furthermore, the powder densification in pellet form or the hybridization with polymer monolith [36,37] will serve as an excellent alternative to enhancing the chemical and thermal stability of tin-based HP-UMPFs. A rating of “4A” appears prerequisite for any open framework that seeks applicability in radioanalytical separation. 

## 4. Conclusions

A quantitative description of macroscopic leaching behavior and the concomitant bulk phase and surface structure transformation of archetypical HP-UMPFs has uncovered the following bullet points itemized as: (a) a triple progressive degradation is roughly regarded as the disassembly of each hierarchy, presumably driven by constant nucleophilic attack and the minimization of surface free energy; (b) both the bulk phase and surface structures have sustained the hydrochloric acid leaching without suffering great degradation of porous texture; (c) the dynamic balance between exposure of confined phosphonate ligands and elimination of free phosphonate ligands largely dictates the remaining functionality during corrosion, achieving real-time tuning cation selectivity towards radionuclide; (d) the effect of acidity on incongruent dissolution prevails over the influence of temperature, while the local ordering leads to a rapid kinetic stability in relevant to amorphous counterparts. The reinforcement of hydrolytic stability and the optimization of ion selectivity under harsh acidic media concurrently remains a challenge for radionuclide separation materials. This plausible degradation mechanism will afford a paradigm for the design of HP-UMPFs that enables the two crucial aspects together.

We are pursuing the line of controlled fabrication of HP-UMPFs with abundant free phosphonate ligands, aiming to establish an efficient radioanalytical separation protocol for tetravalent actinides. However, beyond this scope, the actinide-loaded HP-UMPFs can be envisaged as a precursor to fabricating ternary metal phosphide, displaying great potential in the field of electrochemical water splitting [38], and catalytic transformation of oxygenated hydrocarbons [39]. A cradle-grave-rebirth atlas of HP-UMPFs has emerged for versatile applications.

## Supplementary Materials

The following are available online at http://www.mdpi.com/2079-4991/8/3/166/s1, Figure S1: The HRTEM images of Sn-EDTMP 3, Figure S2: The XPS spectra of pristine (black) and leached (red) tin alkylphosphonates with tentative molecular formula, Figure S3: The selected XPS zones of pristine (black) and leached (red) tin alkylphosphonates: (a) Sn-ATMP; (b) Sn-EDTMP; (c) Sn-DTPMP. The structural breakdown of SnP in the presence of HCl is depicted as (d) to illustrate the formation of new species speculated from XPS spectra, Figure S4: Acidity dependence of SnP dissolution rates and the variation of log Ri versus the reciprocal acidity. (a) tin; (b) phosphorus, Figure S5: Empirical models that describe the leached elemental concentration (*c*) as a function of time (*t*) has been utilized to fit the dissolution kinetics of tin alkylphosphonates: (a) the fitting of tin release; (b) the fitting of phosphorus release, Figure S6: Temperature dependence of SnP dissolution rates and the variation of ln Ri versus the reciprocal temperature. (a) tin; (b) phosphorus. Figure S7: The wide-angle diffraction patterns of Sn-EDTMP; Table S1: The congruent ratio, *r* values of tin alkylphosphonates; Table S2: The chemical, thermal stability of selected porous coordination polymers for radionuclide sequestration.
