# Supplementary material for: The Hydrolytic Stability and Degradation Mechanism of a Hierarchically Porous Metal Alkylphosphonate Framework
Source: Nanomaterials (Basel). 2018 Mar 14;8(3):166. doi: 10.3390/nano8030166 (PMC5869657; doi:10.3390/nano8030166)
Supplement: Supplementary file 1 [file nanomaterials-08-00166-s001.pdf]

The Hydrolytic Stability and Degradation Mechanism of a Hierarchically Porous Metal Alkylphosphonate Framework

Kai Lv *, Chu-Ting Yang, Yi Liu, Sheng Hu and Xiao-Lin Wang

Radiochemistry Lab, Institute of Nuclear Physics and Chemistry, China Academy of Engineering Physics, P.O. Box 919, Mianyang 621900, China; yangchuting@caep.cn (C.-T.Y.); arisewing@126.com (Y.L.); husheng205@caep.cn (S.H.); xlwang@caep.cn (X.-L.W.)

* Correspondence: lvkai@caep.cn; Tel.: +86-816-248-4289

Supplementary Materials:

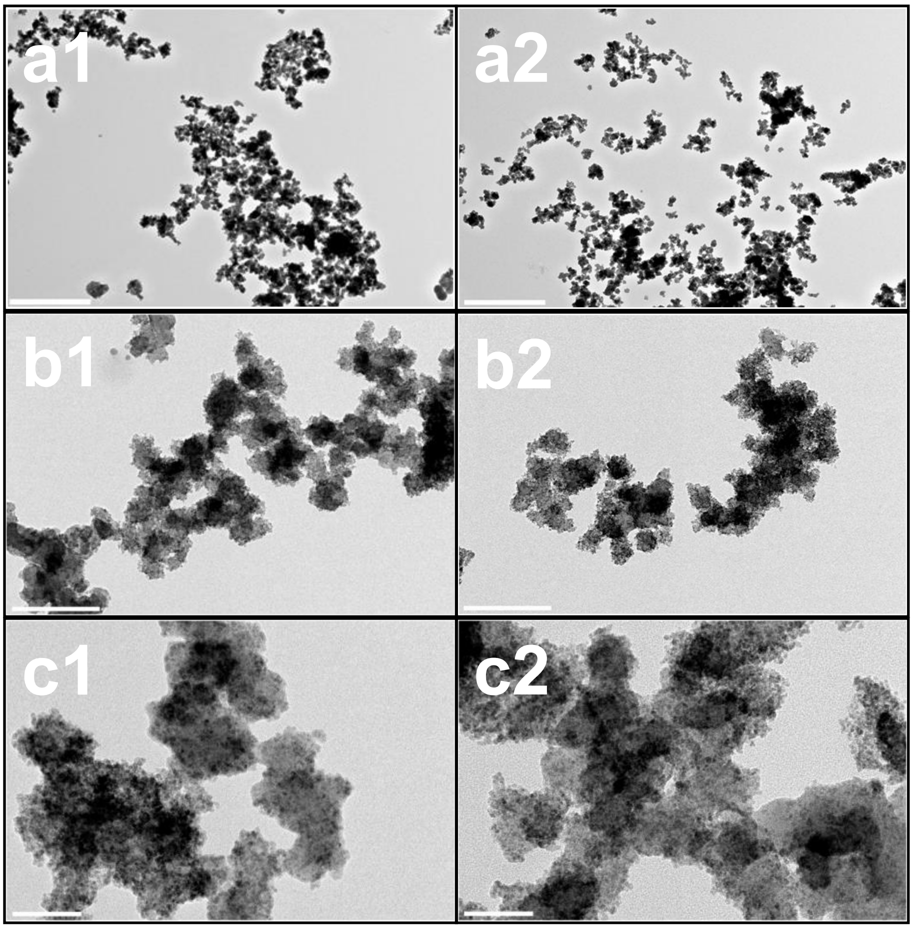

Figure S1. The HRTEM images of Sn-EDTMP 3. The scale bar of a, b, c are 1 μm, 200 nm and 50 nm, respectively. The left, right column of images represents pristine and leached Sn-EDTMP 3, respectively.

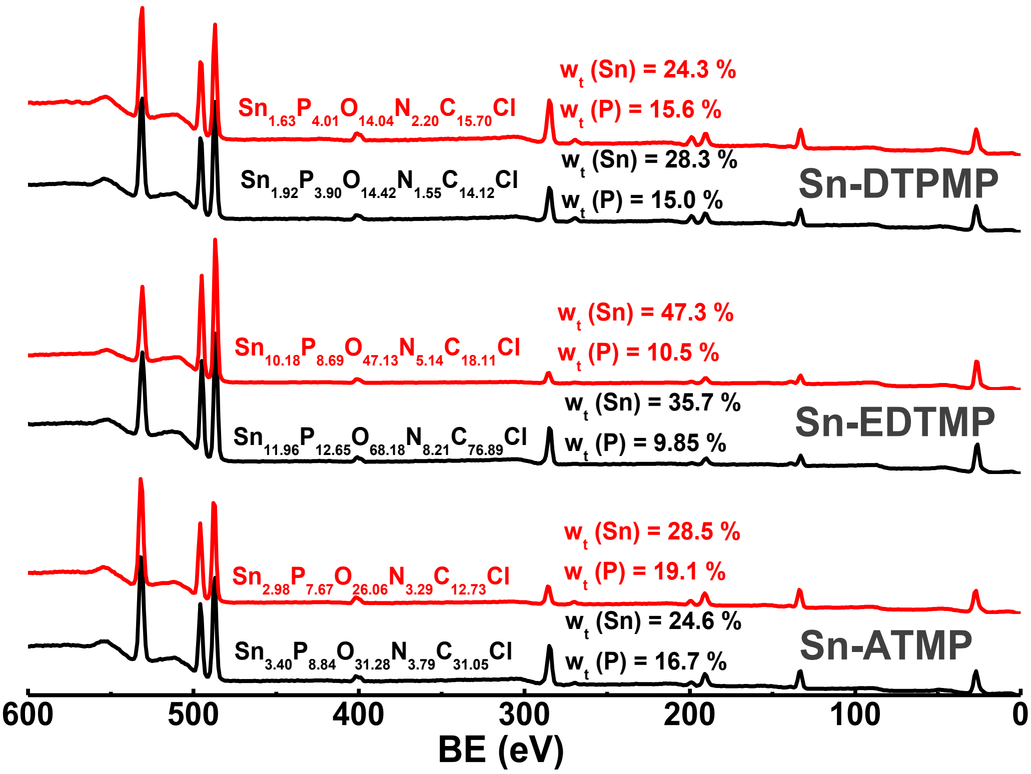

Figure S2. The XPS spectra of pristine (black) and leached (red) tin alkylphosphonates with tentative molecular formula. The weight percentages of tin, phosphorus before and after leaching are demonstrated for comparison.

Table S1. The congruent ratio, r values of tin alkylphosphonates

**Parameter \ Category**		**Sn-HEDP**	**Sn-ATMP**	**Sn-EDTMP**	**Sn-DTPMP**
**Acidity (molL^{-1})**	**0.01**	2.74199	0.99899	0.44533	0.26044
	**0. 1**	2.71802	0.91594	0.44638	0.03086
	**1.0**	3.2708	28.1491	0.08904	4.01853
	**3.0**	3.24644	4.05557	0.41856	3.3349
**Temperature (K)**	**303**	4.22529	5.51507	0.06174	5.61172
	**318**	4.41881	5.31045	0.07381	5.69931
	**333**	4.73241	5.50817	0.1434	5.44445

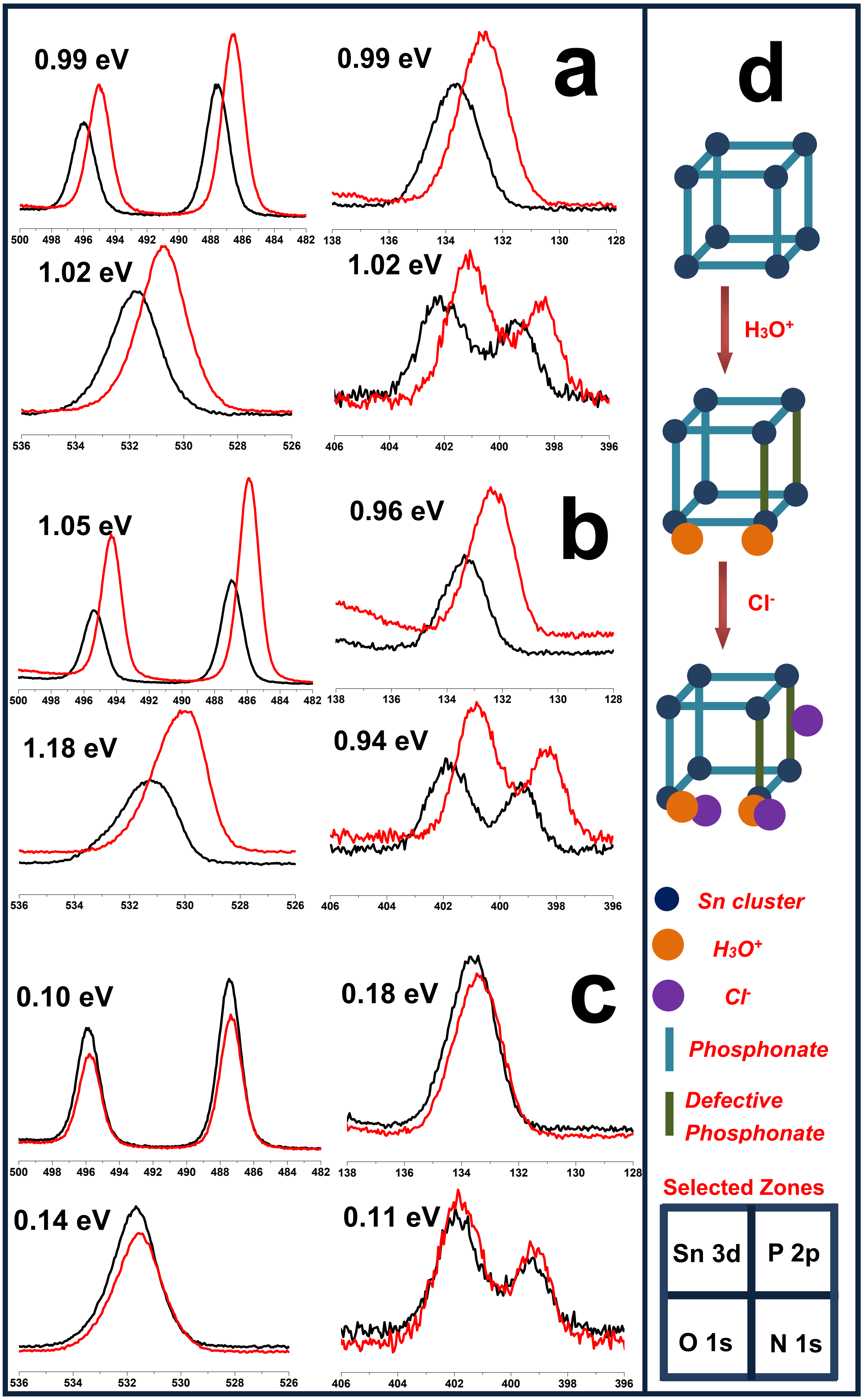

0.99 eV
0.99 eV
a
1.02 eV
1.02 eV
1.05 eV
0.96 eV
b
1.18 eV
0.94 eV
0.10 eV
0.18 eV
c
0.14 eV
0.11 eV
d
H3O+
Cl-
Sn cluster
H3O+
Cl-
Phosphonate
Defective Phosphonate
Selected Zones
Sn 3d
P 2p
O 1s
N 1s

Figure S3. The selected XPS zones of pristine (black) and leached (red) tin alkylphosphonates: (a) Sn-ATMP; (b) Sn-EDTMP; (c) Sn-DTPMP. The structural breakdown of SnP in the presence of HCl is depicted as (d) to illustrate the formation of new species speculated from XPS spectra.

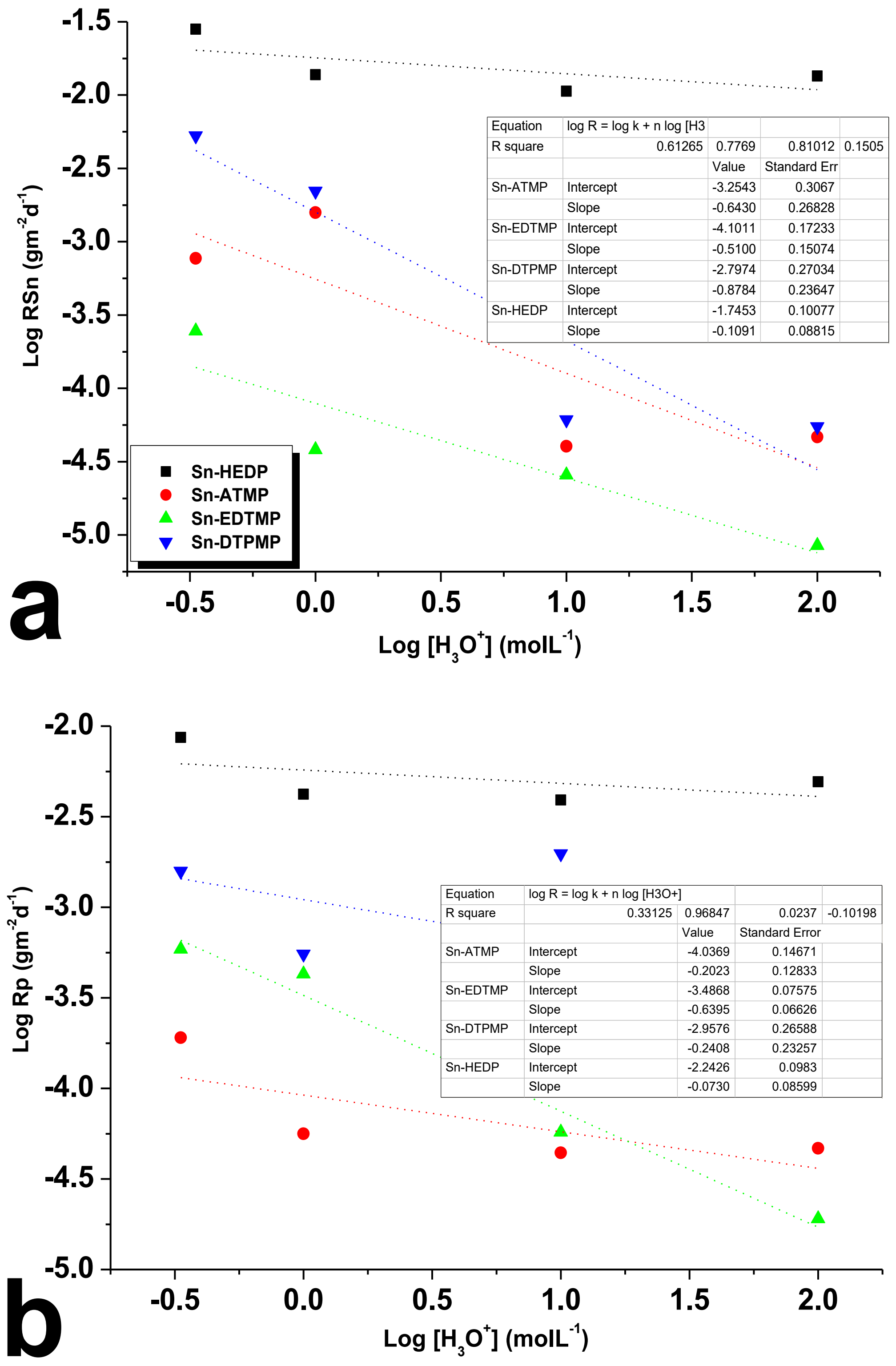

Figure S4. Acidity dependence of SnP dissolution rates and the variation of log R_i versus the reciprocal acidity. The R_i, k, n represent the normalized dissolution rates, apparent normalized dissolution constant, partial order related to proton concentration, respectively. (a) tin; (b) phosphorus.

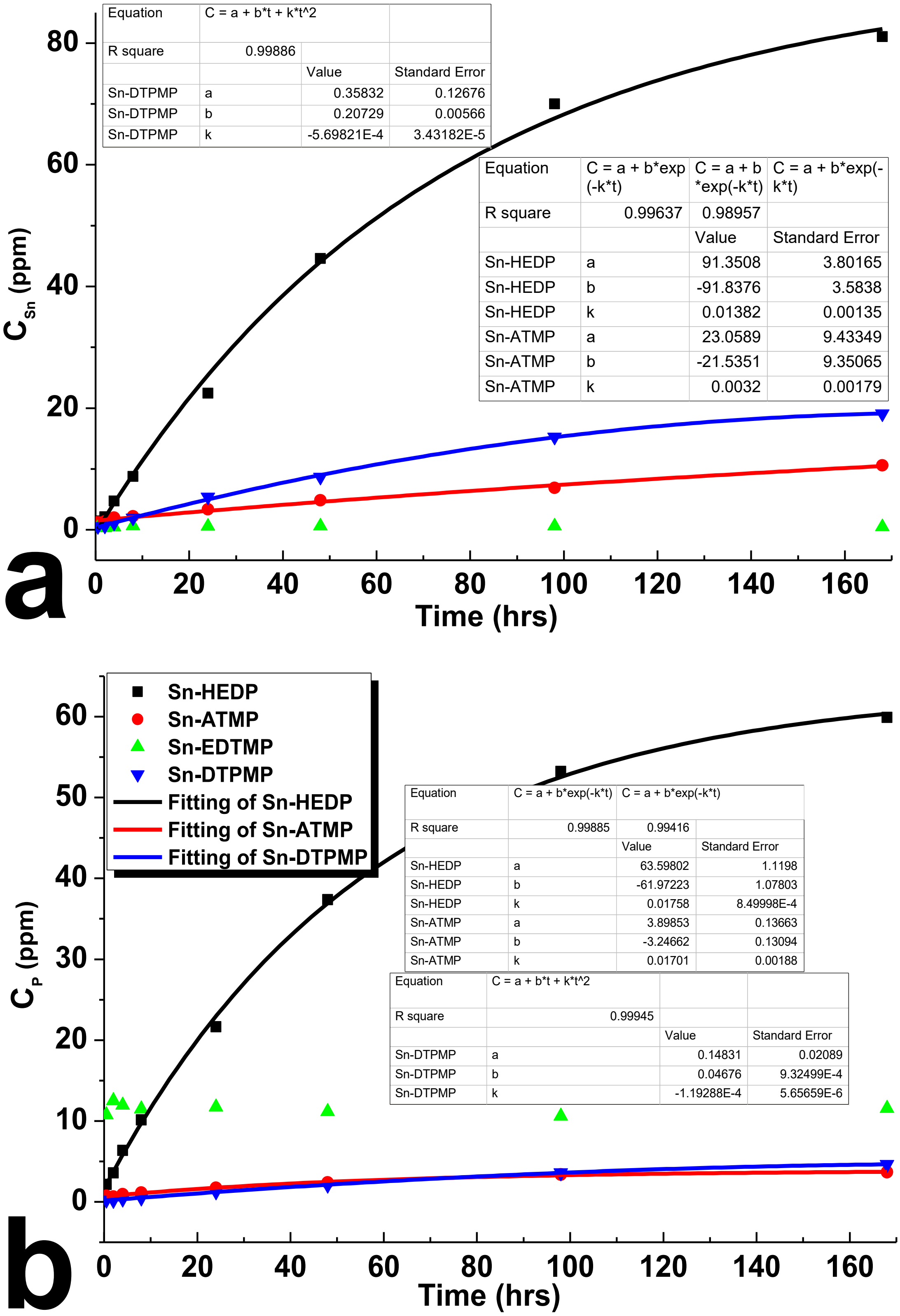

Figure S5. Empirical models that describe the leached elemental concentration (C) as a function of time (t) has been utilized to fit the dissolution kinetics of tin alkylphosphonates: (a) the fitting of tin

release; (b) the fitting of phosphorus release. The first-order equation is: $C = a + b*e^{-kt}$, where a, b is related to the solubility limit of SnP and k is the dissolution rate constant. The parabolic expression is: $C = a + b*t + k*t^2$, where a, b, k are the dissolution parameters. (a) tin; (b) phosphorus.

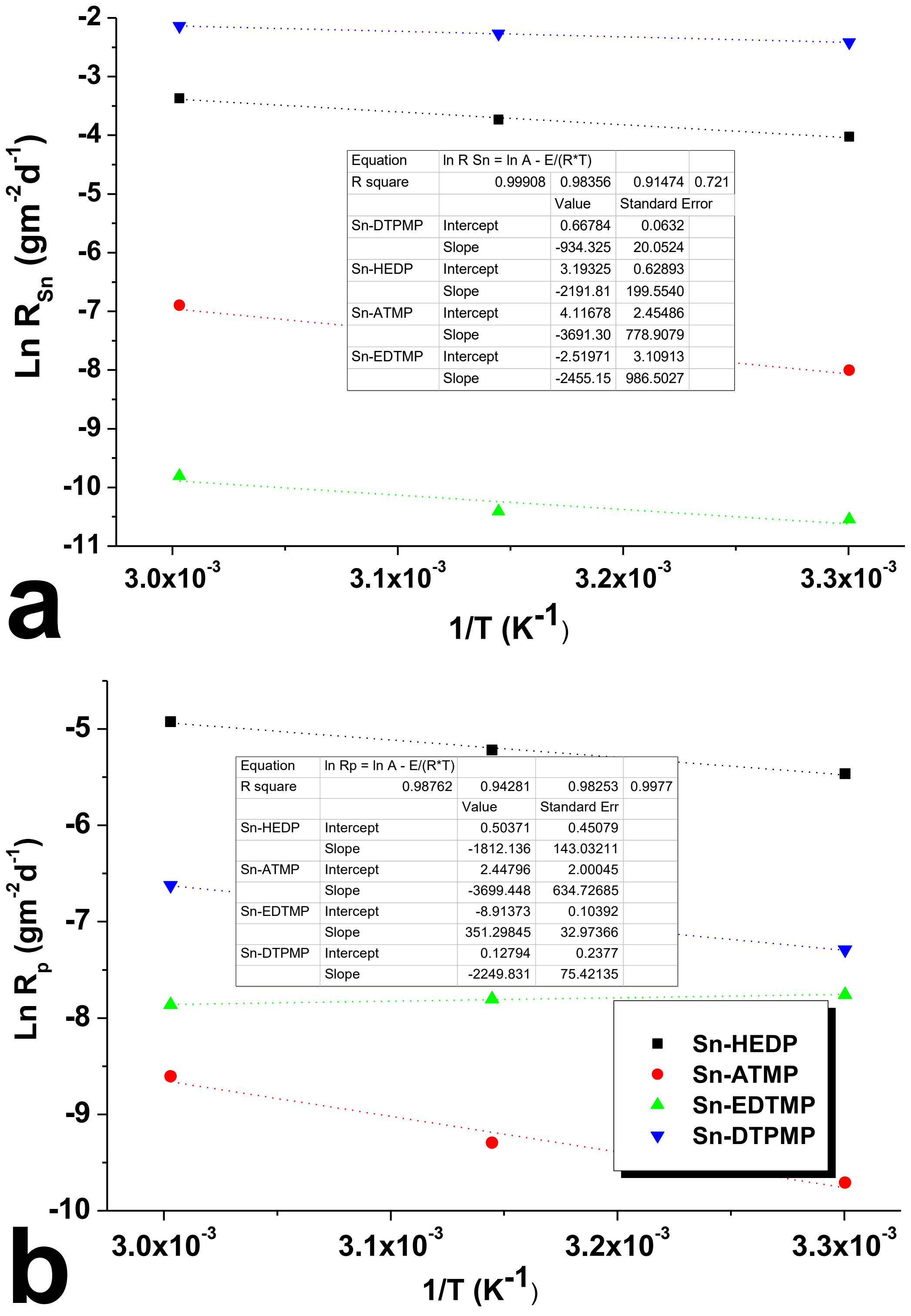

Figure S6. Temperature dependence of SnP dissolution rates and the variation of ln R_i versus the

reciprocal temperature. The R_i, A, E, R represent the normalized dissolution rates, pre-exponential factor equating with dissolution constant, activation energy and the ideal gas constant, respectively. Note that the A value depends solely on the temperature, independent of acidity. The R value is 8.314 $Jmol^{-1}K^{-1}$. (a) tin; (b) phosphorus.

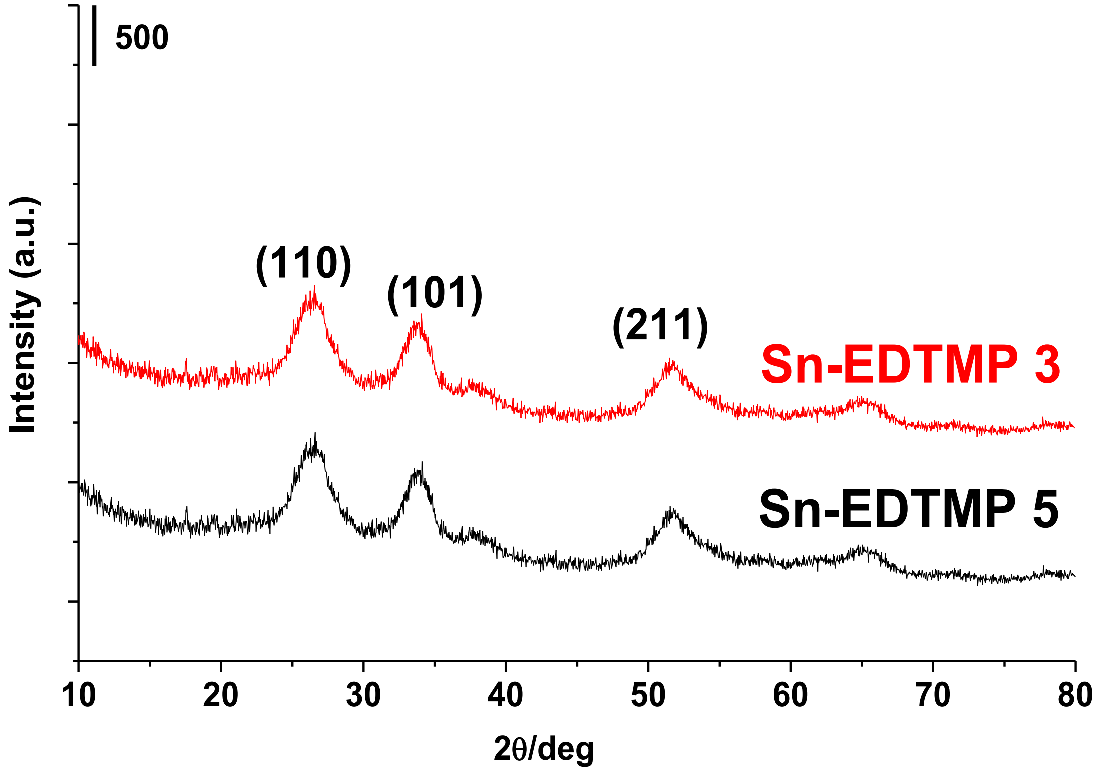

Figure S7. The wide-angle diffraction patterns of Sn-EDTMP

Table S2. The chemical, thermal stability of selected porous coordination polymers for radionuclide sequestration

Sample	Metal	Linker	Category	Thermal (°C)	Chemical	Target	Grade	Ref
UiO-66	Zr	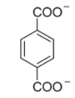	Carboxylate MOFs	430	1 M HCl for 2h	U, Th	4A	1
SZ-1	Zr	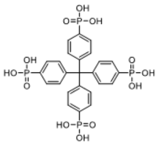	Single-crystal MPFs	210	12 M HCl for 12 h	U	4A	2
H-Sn	Sn	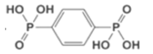	UMPFs	420	NM	Am	NM	3
Sn-EDTMP	Sn	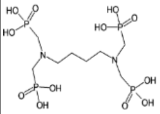	HP- UMPFs	590	3 M HCl for 24 h	U, Th	“4A”[a]	4

NM: not measured
a: retention of porosity but no sign of any loss of bulk periodicity due to amorphous nature

Reference

1 Kandiah M, Nilsen MH, Usseglio S, Jakobsen S, Olsbye U, Tilset M, Larabi C, Quadrelli EA, Bonino F, Lillerud KP. Synthesis and stability of tagged UiO-66 Zr-MOFs. Chem Mater. 2010; 22: 6632-6640.

2 Zheng T, Yang ZX, Gui DX, Liu ZY, Wang XX, Dai X, Liu ST, Zhang LJ, Gao Y, Chen LH, Sheng DP, Wang YL, Diwu J, Wang JQ, Zhou RH, Chai ZF, Albrecht-Schmitt TE, Wang S. Overcoming the crystallization and designability issues in the ultrastable zirconium phosphonate framework system. Nat Commun. 2017; 8: 15369-15379.

3 Silbernagel R, Shehee TC, Martin CH, Hobbs DT, Clearfield A. Zr/Sn (IV) phosphonates as radiolytically stable ion-exchange materials. Chem Mater. 2016; 28: 2254-2259.

4 Lv K, Han J, Yang CT, Cheng CM, Luo YM, Wang XL. A category of hierarchically porous tin (IV) phosphonate backbone with the implication for radioanalytical separation. Chem Eng J. 2016; 302: 368-376.
